# Spinal Cord Stimulation for Freezing of Gait: From Bench to Bedside

**DOI:** 10.3389/fneur.2019.00905

**Published:** 2019-08-27

**Authors:** Erich Talamoni Fonoff, Andrea C. de Lima-Pardini, Daniel Boari Coelho, Bernardo Assumpção Monaco, Birajara Machado, Carolina Pinto de Souza, Maria Gabriela dos Santos Ghilardi, Clement Hamani

**Affiliations:** ^1^Hospital Israelita Albert Einstein, São Paulo, Brazil; ^2^Department of Neurology, University of São Paulo, São Paulo, Brazil; ^3^Laboratory of Integrative Motor Behaviour, Centre for Neuroscience Studies, Queen's University, Kingston, ON, Canada; ^4^Human Motor Systems Laboratory, School of Physical Education and Sport, University of São Paulo, São Paulo, Brazil; ^5^Biomedical Engineering, Federal University of ABC, Santo André, Brazil; ^6^Neurosurgery, Association for Assistance of Disabled Children (AACD), São Paulo, Brazil; ^7^Division of Neurosurgery, Harquail Centre for Neuromodulation, Sunnybrook Research Institute, University of Toronto, Toronto, ON, Canada

**Keywords:** spinal cord stimulation, gait, Parkinson's disease, pain, freezing of gait

## Abstract

Spinal cord stimulation (SCS) has been used for the treatment of chronic pain for nearly five decades. With a high degree of efficacy and a low incidence of adverse events, it is now considered to be a suitable therapeutic alternative in most guidelines. Experimental studies suggest that SCS may also be used as a therapy for motor and gait dysfunction in parkinsonian states. The most common and disabling gait dysfunction in patients with Parkinson's disease (PD) is freezing of gait (FoG). We review the evolution of SCS for gait disorders from bench to bedside and discuss potential mechanisms of action, neural substrates, and clinical outcomes.

## Introduction

Spinal cord stimulation (SCS) has been used for several decades as a minimally invasive neuromodulation strategy for the treatment of patients with chronic pain (1). With a good efficacy profile and a relatively low incidence of side effects, SCS comprises one of the proposed therapeutic modalities in guidelines for the management of refractory neuropathic pain (2). In recent experimental work, SCS has also been suggested to improve motor and gait dysfunction in parkinsonian states (3, 4). In Parkinson's disease (PD), a common and disabling problem is freezing of gait (FoG). Although in its infancy, recent studies using SCS for the treatment of FoG have shown promising results (5–8).

In this review, we first describe particular aspects of FoG that pose challenges for the development of therapeutic interventions and the interpretation of post-treatment results, including its complex mechanisms, episodic nature, and multifactorial pathophysiology. We then summarize experimental and clinical data. Finally, we analyze anatomical and physiological concepts that may assist in the development and or improvement of SCS strategies to treat gait dysfunction and FoG. The search strategy on PubMed included the following terms: SCS OR dorsal column stimulation AND Parkinson, which retrieved 126 abstracts. Twenty one were directly related to the topic. Those articles were used as a starting point for the search of additional, related articles that would bring relevant clinical data, cases, and series reports.

## Freezing of Gait: a Puzzling Phenomenon

**Figure 1:**
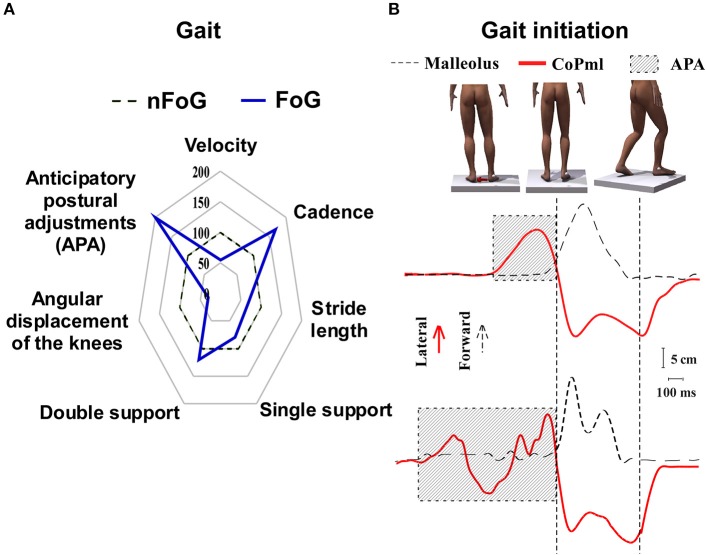
Characteristic motor patterns associated with freezing of gait in Parkinson's disease. **(A)** Patients exhibit dysfunction prior to or during freezing of gait. Note that these variables are recorded in different tasks: impairment of APA occurs prior to step initiation; altered cadence and stride length during gait, and disorders of angular displacement of the knee during the so-called trembling knees. The central dotted line of the satellite plot represents patients without freezing of gait (nFoG) data. Deviations along the axes radiating from the center of the plot represent the percentage in which patients with freezing of gait (FoG) differ from nFoG (blue line). **(B)** Representation of the step initiation task. The panel on the top represents the sequence of the events during step initiation. The red arrow shows the shifting of body weight before moving the opposite foot forward. The curves on the middle and bottom show the CoPml (red curve) and malleolus displacement (dotted line). The upper curves represent a normal stepping eliciting one APA before the leg movement. The lower curves represent multiple and longer APAs usually seen during FoG episodes. The hatched area are APAs (time from the increasing of mediolateral force to the step onset).

Of all motor and non-motor symptoms in PD, FoG is one of the most incapacitating and enigmatic. It affects nearly 50% of moderate idiopathic PD patients and 80% of subjects in more advanced stages of the disease (9). In general, FoG may be defined as a transitory impossibility to keep the progression of gait despite the intention to walk (10). FoG is a major risk factor for falls (11), significantly contributes to functional incapacity (12), and frequently leads to a reduction in quality of life (13). Factors that trigger and relieve FoG suggest that this is a complex entity with multiple interconnected mechanisms. FoG mostly occurs during walking through narrow passages (14), situations of cognitive overload (e.g., dual tasks) (15), anxiety (16), and turning movements (17). Factors that alleviate freezing are certain visual patterns (e.g., stripes on the floor) (18), auditory cues (19), proprioceptive and haptic stimuli (20), and other compensation strategies (21). The pathophysiology of FoG comprises an interplay of heterogeneous sensory, motor, and cognitive aspects and remains poorly understood. Compared to non-freezers, PD patients with FoG experience more pronounced postural instability and impaired gait (22). In FoG patients, gait features are significantly impaired compared with control patients without FoG (nFoG), representing a global pattern of gait impairment. Changes in motor patterns prior to freezing include a higher cadence, a smaller stride length (23, 24), and dysfunctional anticipatory postural adjustments (APA) (25) (Figure 1A). APA dysfunction occurs especially in patients with start hesitation, characterized by a difficulty in step initiation. The transition between the upright stance to movement that occurs during step initiation is challenging, given that forward movements are a source of body disequilibrium. APA is required to counterbalance the internal forces generated to move the center of mass forward, allowing for a controlled step initiation. Prior to step initiation, APA is usually characterized by a sequence of events beginning with a backward displacement of the center of pressure toward the moving leg. Thereafter, the center of pressure is displaced toward the supporting foot. The mediolateral component is thought to be involved in balance control, while the sagittal component enables the forward acceleration of the center of mass (26). Although mechanisms of APA are not completely understood, adjustments are modulated by higher brain centers, such as the supplementary motor area (SMA) (27, 28). APA abnormalities restrain the body weight shift, leading to shorter steps with smaller amplitudes. Patients with start hesitation have multiple (29) and impaired APAs (30), which could lead to a hesitant ineffective initiation of gait (Figure 1B).

Pre-clinical studies investigating mechanisms of freezing and gait dysfunction highlight changes in subcortical and brainstem circuits, including the mesencephalic locomotor region (MLR) and the pedunculopontine nucleus (PPN) (31, 32). Following 1-methyl-4-phenyl-1,2,3,6-tetrahydropyridine (MPTP) administration, ~50% of non-human primates developed FoG (33). In naïve animals, deficits produced by MLR lesions mimic those observed in parkinsonian states (34). Stimulation of the MLR exerts complex effects. Depending on the stimulation site within the PPN and frequency, it may augment or reduce FoG (35). From a translational perspective, observations from non-humans primates must be considered with caution. Postural adjustments are pivotal for an efficient biped gait in humans, while non-human primates often express quadruped locomotion. In order to keep balance during bipedal stance, humans require more intricate postural adjustments that probably involve a more complex neural circuitry. This may help to elucidate discrepancies between clinical and pre-clinical models and explain why studies aiming to clarify mechanisms of FoG are often more elucidative than studies in experimental models.

**Figure 2:**
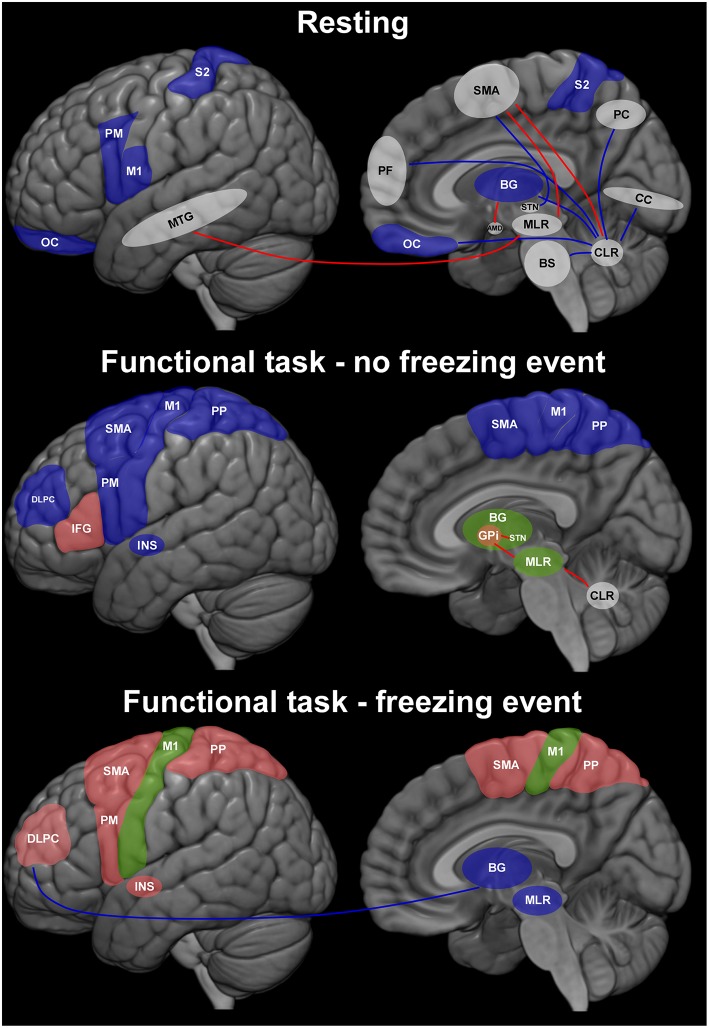
Representation of brain dynamics in three conditions during which patients with Parkinson's disease (PD) were assessed. The results describe the contrast between patients with or without freezing of gait (FoG+ > FoG–). Blue regions are those for which available evidence shows less activity in FoG+ than in FoG–; green indicates regions with higher and lower activity in FoG+; red represents regions in which activity was higher in FoG+ than FoG–. Traces indicate connections between two regions (red: higher; blue: lower in FoG+). White regions are those involved in brain circuits (connectivity studies) without representation of level of the activity between FoG+ and FoG–. AMD, amygdala; BG, basal ganglia; BS, brainstem; CC, calcarine cortex; DLPFC, dorsolateral prefrontal cortex; GPi, internal globus pallidus; IFG, inferior frontal gyrus; INS, insula; M1, primary motor cortex; MLR, mesencephalic locomotor region; MTG, middle temporal gyrus; OC, orbitofrontal cortex; PC, precuneus; PF, prefrontal cortex; PM, premotor cortex; PP, posterior parietal cortex; S2, secondary somatosensory cortex; SMA, supplementary motor area; STN, subthalamic nucleus.

In PD patients, comparative functional studies using positron emission tomography, single-photon emission computed tomography, functional magnetic resonance imaging, and functional near infrared spectroscopy have been conducted at rest and when functional tasks were performed in the absence or presence of freezing with intriguing results (Figure 2). At rest, patients with FoG (FoG+) showed decreased activation of the orbitofrontal cortex, premotor cortex (36, 37), and basal ganglia (38) compared to patients who did not experience FoG (FoG-). Patients with FOG+ had increased functional connectivity (FC) between frontal areas, particularly the SMA, the cerebellar locomotor region (CLR) and MLR. In contrast, these patients had decreased FC between the prefrontal cortex and basal ganglia (39). Interestingly, freezers showed decreased structural and functional connectivity between SMA and subthalamic nucleus (STN), known to be involved in the inhibition control (40). The cerebellum, more specifically the dentate nucleus, had decreased connectivity with brainstem, basal ganglia, frontal, and parieto-occipital cortices in FoG+ compared to FoG- (41). Additional findings in FOG+ were increased FC between the putamen and amygdala (42), and between the MLR and middle temporal gyrus (MTG) (43). It is noteworthy the increased interaction between areas that process movement planning (SMA), emotion (amygdala), and sensory integration (MTG) with subcortical regions associated with the processing of movement initiation (CLR and MLR). This highlights the contribution of subcortical structures that process emotional and sensory information, probably activating regions involved in motor planning and gait initiation.

Distinct brain activity has been found on imaging studies depending on whether freezing episodes were present during task performance. In the absence of freezing, a decrease in frontal activity has been demonstrated along with an inconsistent activation of subcortical regions (44–47).

During the occurrence of freezing, studies have shown less subcortical and sensorimotor cortical activity (48, 49), but higher activation of frontal regions (50, 51) and insula (50). A decrease in functional connectivity between the cognitive network (DLPFC and posterior parietal cortex) and basal ganglia (49) was correlated with increased frequency of FoG episodes during a virtual pedaling task.

These findings point to a dynamic profile of brain correlates of freezing, evidencing a contribution of frontal areas and the reduced participation of sensorimotor cortex, basal ganglia, and brainstem during motor arrests. However, caution is needed when interpreting the above-mentioned studies due to the use of distinct approaches (e.g., imagined gait, cycling, and manual tasks). Also, in most neuroimaging studies patients were lying in the scanner without the requirements of bipedal postural control. Another important limitation is the incomplete information provided by such studies on the characteristics of freezing (start hesitation, turning, during gait), which may have different pathophysiological mechanisms. This substantially increases variability.

In addition to imaging studies, brain networks involved in freezing have also been investigated with *in vivo* electrophysiology and non-invasive wireless scalp EEG. Tard et al. (52) recorded abnormal beta band oscillations in central and frontal areas associated with a disruption in the integration between attention patterns frequently found during auditory task and motor preparation in FoG+ patients. As scalp EEG renders access mostly to convexity neocortical areas, it has been used to study the correlation between this episodic phenomenon and cognitive networks. Butler et al. (53) showed an excessive recruitment of lateral premotor areas and the loss of automatic motor control related to attentional deficits associated with FoG. Other studies have shown that specific patterns of scalp EEG may be used to identify and even predict FoG episodes (54).

In addition to brain circuits, those in the spinal cord have also been associated with disrupted gait control in FoG+. The normal gait should integrate feed-forward information processed in cortical control centers, basal ganglia, cerebellum, and brainstem and feedback input derived from the periphery to modulate spinal patterns generation centers (CPG) (55). Although CPGs are capable of generating complex patterns, such as autonomous gait, they receive extensive connections from higher brain centers that generate motor engrams for volitional or reactive behavior. Gait as a complex behavior is generated by the interaction between brain circuits and CPGs mediated by intricate mechanisms of descending feed-forward control and feedback loops. These comprise pathways that control sensory information, posture, and balance, including cerebellar, vestibular, and reticular systems. FoG may occur when brain circuits that should integrate multifactorial stimuli in higher brain circuits are not capable of processing sufficient information for a timely convergence into the complex behavior of walking. Gait initiation requires processing and coordination of updated environmental information with exact coupling of postural adjustment in advance of steps forward (56). This mechanism seems to be disrupted in PD patients with FoG (57). For example, during step initiation there must be an efficient pairing between the preparation phase and voluntary step, which is modulated by the SMA (27). Defective APAs or disengaged postural corrections of steps have been directly related to the occurrence of FoG (29). Our group has recently found that SCS was able to decrease the duration of FoG and the timing between APA and step initiation in severe freezers (58). One hypothesis is that, by activating ascending spinal pathways that reach the SMA, high frequency SCS (300 Hz) might have corrected dysfunctional postural adjustments, improving gait and FoG (59). This is in agreement with clinical neuroimaging data and pre-clinical electrophysiology studies suggesting that SCS modulates sensorimotor, prefrontal, cingulate, and insular cortices (3, 4, 60, 61), all regions considered to play a role in mechanisms of FoG (3, 4, 60–62).

## Translational Helix: Concepts From the Bench to the Bedside

**Figure 3:**
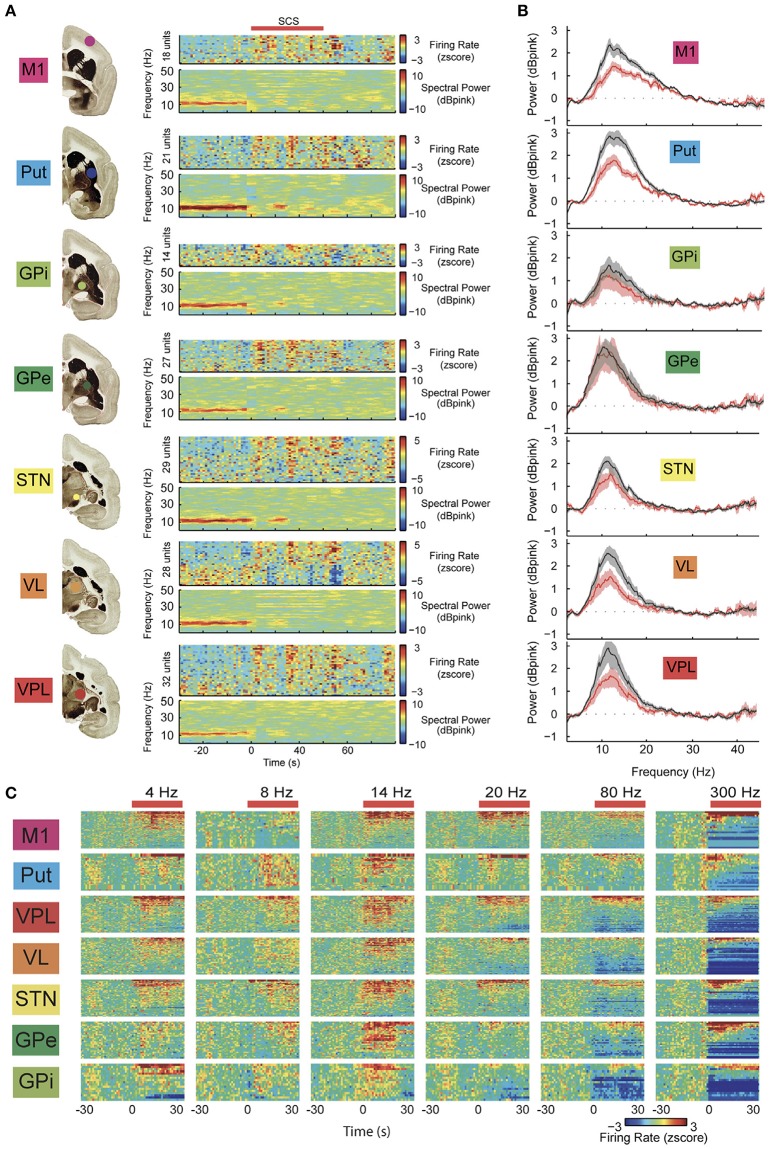
**(A)** Example of parallel changes in local field potential (LFP) power and neuronal firing rate in multiple structures of the cortico-basal ganglia-thalamic loop during high frequency spinal cord stimulation (SCS). Note the immediate reduction of low-frequency oscillations (beta band) in response to SCS (red bar, stimulation frequency: 4 Hz; color codes denote decibels above pink noise background for LFPs). **(B)** Average LFP spectra for all recording sessions normalized to pink noise showing a significant SCS-induced reduction in LFP beta-power in all structures, except the globus pallidus externus (GPe). Shaded area denotes 95% CI with 100 bootstraps. **(C)** Standardized neuronal firing rate response to different SCS frequencies in multiple structures of the basal ganglia circuits (neurons rank ordered according to responses). Note that most significant changes in neuron firing were achieved at higher frequencies. M1, primary motor cortex; Put, putamen; VPL, thalamus ventroposterior nucleus; VL, thalamus ventrolateral nucleus; STN, subthalamic nucleus; Gpi, globus pallidus internus. Adapted with permission from Santana et al. (3) (Figures 2A,B, 3A).

Although SCS has been used in the past for the treatment of various movement disorders (63), its popularity in the last two decades have faded. Potential reasons include the lack of consistent and reproducible results, limited knowledge on its mechanism of action and technological restrictions. This began to change in 2009, when Fuentes et al. showed that SCS applied to dopamine-depleted mice resulted in a remarkable improvement in locomotion (4). Possible explanations for this finding were the modulation of oscillatory brain activity and the fact that the spinal cord is a major channel of afferent information to the brain (59). Strikingly, locomotive behavior initiated a few seconds after stimulation onset and proceeded by instantaneous changes in local field potentials (LFP) in the motor cortex and striatum (4). The proposed mechanism to mediate this effect was the inhibition of pathological synchronized slow wave oscillations often found in motor circuit related structures of PD patients and animal models (3–5). While stimulation induced a prompt shift from lower to higher frequencies in motor circuits, this tended to outlast SCS discontinuation by up to 50 s, suggesting a significant carry-over effect. In non-human primates, stimulation parameters that induced changes in kinematic measures were also able to effectively change oscillatory patterns in thalamo-cortical-basal ganglia networks (Figure 3) (3). Similar to the benefits described above, gait dysfunction in PD was shown to be improved in patients treated with upper thoracic cord SCS at high frequencies (e.g., 300 Hz), with a carry-over effect being clearly noted (6, 58). Regarding electrochemical interactions, in dopamine transporter knockout mice (DAT-KO) the dose of L-dopa required to induce locomotion was decreased to one fifth following SCS (4). In contrast, synergistic effects between dopaminergic medications and SCS have not been clinically documented.

### Relevant Anatomical Aspects of the Spinal Cord in the Context of SCS

**Figure 4:**
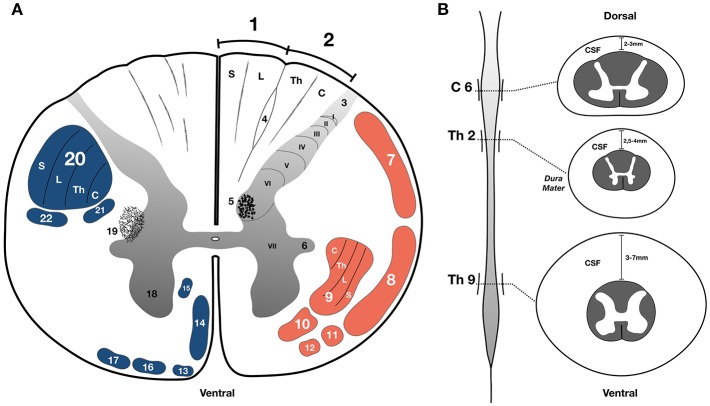
**(A)** Schematic illustration of a transverse section of the thoracic spinal cord; the upper part of the figure shows the position of structures in white (white background) and gray matter (gray gradient); 1. Gracile fasciculus of Goll; 2 Cuneate fasciculus of Burdarch (dorsal column); 3. Lissauer's tract; 4. Semilunar tract (Schultz's comma); 5. Thoracic nucleus of Clarke; 6. Intermediate column; 7. Posterior spinocerebellar tract; 8. Anterior spinocerebellar tract; 9. Lateral spinothalamic tract; 10. Anterior spinothalamic tract; 11. Spino-olivary tract; 12. Spinotectal tract; 13. Tectospinal tract; 14. Anterior corticospinal tract; 15. Reticulospinal tract; 16. Anterior vestibulospinal tract; 17. Olivo-spinal tract; 18. Anterior column (Gray matter); 19. Reticular formation of the spinal cord; 20. Lateral corticospinal tract; 21. Lateral vestibulospinal tract; 22. Rubro spinal tract; Lamina of Rexed (I to X–gray matter). **(B)** Representation of the spinal cord in longitudinal axis showing the cervical and lumbar enlargements and the respective transverse sections and the distance from the dura to the surface of the cord at different levels. Somatotopy abbreviations; C, cervical; Th, thoracic; L, lumbar; S, sacral; C6, sixth cervical level; Th2, second thoracic level; Th9, ninth thoracic level; CSF, cerebrospinal fluid. Adapted with permission from de Souza et al. (68).

Spinal cord stimulation (SCS) has been used for many years with a relatively low profile of adverse events. This is probably due to the fact that electrodes are implanted in the epidural space underneath the laminae and spinous processes. As electrical stimulation is routinely delivered to the posterior aspect of the cord in therapeutic SCS protocols, most of the current invariably spreads to the dorsal columns and occasionally posterior radiculi. These elements are mainly composed by thick myelinated axons that are excited at low thresholds and may detour electrical current due to reduced impedance of its fibers. However, in different spinal cord levels there are also different fiber content which vary in diameter and consequently in electrical excitation threshold. Within the cervical spine enlargement, there are vast numbers of sensory fibers coming from the upper limbs, as well as internuncial and second order neurons. On the other hand, at mid and upper thoracic levels, the cord is considerably thinner for two main reasons: (i) a smaller contingent of segmental afferents coming from less densely innervated dermatomes in the torso and (ii) long projection axons that tend to progressively decrease in diameter after entering the cord in the dorsal root entry zone (64). At theses levels, the propagation velocity is decreased while the stimulation threshold in the dorsal column is increased (65). In addition, ascending fibers from lower limbs course medially in these spinal levels, occupying a deeper position in the dorsal columns. Thus, SCS applied in high thoracic cord is more likely to modulate deeper fiber layers and dorsal horn before generating intense lower limb paresthesias (66). As an example, at spinal thoracic levels the posterior thoracic nucleus (Clarke's column) located in the depth of gray matter of the dorsal horn (lamina VII of Rexed) gives origin to important ascending fibers. This nucleus is a major relay center for unconscious proprioception with cells that collateralize and send afferents within the dorsal column and spinocerebellar tracts (67) directly reaching various structures in the brainstem, diencephalon and deep cerebellar nuclei. In upper thoracic levels, where the cord is thinner, most long projection fibers are composed of small diameter fibers when compared to those at the spinal enlargements (Figure 4). Apparently, the practical result of this is that SCS at this level can reach a wider range of ascending tracts with similar stimulating thresholds.

### Mechanistic Hypotheses

At a first glance, it may seem somewhat evident that SCS would improve gait directly by facilitating local spinal circuits directly in charge of limb muscle control. Although there might be a local component, as SCS induces improvement in gait performance (e.g., stride length, velocity) the improvement in FoG, which is mediated mainly by brain circuit dysfunctions, suggests that the effect of SCS is more likely to occur on suprasegmental circuits though the stimulation of ascending fibers. In support of this hypothesis, robust inhibition of parkinsonism-related slow wave brain oscillations has been demonstrated in rodent and primate PD models (3–5, 59). However, the percentage of fibers or which ascending systems should be excited to induce this effect remain unclear. Unfortunately, none of the pre-clinical studies discussed this topic in detail, probably because epidural SCS applied to small animals is rather unselective due to diminutive dimensions of the cord. So far, the dorsal columns were picked as natural candidates to be involved in this effect. They are the most superficial and probably have the lowest threshold for epidural SCS. However, data from recent clinical studies suggest that the most efficient stimulation parameters reach deeper sites in the spinal cord (6) or are more comprehensive (less selective). For instance, when SCS was applied deeper into the upper thoracic cord due to the steering of electrical field (see technological and technical issues), it excites the dorsal columns but probably also a greater variety of ascending afferents and long propriospinal fibers located adjacent to the gray matter of the dorsal horn. Additionally, when SCS is applied to the lower thoracic spinal cord, the most efficient parameters include long pulse widths, with lower frequencies tested so far (7). These are in line with the current hypothesis that therapeutic SCS for gait should include multiple projection bundles to brainstem, cerebellum, basal ganglia, thalamus, and cortical areas, besides acting on local and integrated spinal circuits. Among various cortical areas that probably mediate SCS effects on gait, dysfunctions in SMA are directly involved in the pathophysiology of FoG. It has been found that SCS may influence neuronal firing in the SMA, a key hub for controlling gait initiation (27, 64). The SMA does not receive direct thalamic projections but it does receive inputs from somatosensory regions (SI, SII, and area 5) (65). In fact, our recent study showed that SCS improved the timing of APAs during gait initiation (58), a behavior found to be modulated by SMA (27). As mentioned above, frontal activity (e.g., SMA) is increased, whereas subcortical and sensorimotor cortex activity is decreased during motor arrests (48, 50). Altered activity of SMA could intensify its influence over the subthalamic nuclei (STN) via the hyper-direct pathway (66). The increased STN firing in PD states could influence the globus pallidus internus, inhibiting thalamo-cortico-basal circuit activity (66), while reducing activity in the mesencephalic motor area and PPN (67). By inhibiting pathological synchronized slow brainwave oscillations in the SMA, SCS could restore physiological aspects of neuronal circuits known to be involved in gait initiation.

#### Technological and Technical Issues

Bearing the anatomical and electrodynamic features of spinal cord elements in mind, electrode configuration and stimulation field become important variables. Electrodes that provide two or more parallel columns of stimulation contacts and allow multiple combinations of settings are more versatile. Paddle electrodes with three columns of contacts may offer some advantages, since they allow the correction of lateral shift and facilitate the appraisal of the physiological midline. Although, this montage may also be achieved with percutaneous electrodes, at least three leads have to be implanted in order to provide similar coverage. Also useful is the transverse tripolar montage with a middle cathode sided by a pair of anodes. This configuration prevents afferent radiculi from unwanted stimulation, while steering the electrical field further into deep spinal cord elements (69). Transverse stimulation also tends to be more selective than monopolar or longitudinal bipolar stimulation (70) and has been associated with promising clinical results (6).

Percutaneous leads are quite popular among pain physicians because they allow electrode implants to be performed through a puncture. Also, they can be inserted into just about any spinal level and travel longitudinally to the first segments of cervical (71). Those leads are mainly implanted in lower thoracic levels for the treatment of pain in the lower limbs and low back (72–77). The method was applied in most anecdotal reports serendipitously describing improvement in PD symptoms. The larger series reported to date followed this classical method, implanting percutaneous leads over the lumbar spinal cord enlargement (7, 8, 78). The best parameters for treating PD symptoms in that study included a relatively long pulse width (PW). Although this does not necessarily deliver more energy, currents applied for a longer time allow slow depolarizing ion channels in dendrites, cell bodies, and in lesser diameter and poorly myelinated axons to be excited (79). Also, a denser stimulation will recruit elements located deeper in the spinal cord (80), possibly including gray matter regions and projection fibers located in quadrants of the cord other than the dorsal columns (Figure 4A). Conversely, these parameters increase the chance of direct stimulation of nerve rootlets, which may cause discomfort in adjacent dermatomes or muscle contractions in correspondent myotomes (81).

The frequency of stimulation may also change responses from the neural tissue. In the spinal cord, low frequency stimulation often induces intermittent paresthesias or a sense of vibration, while frequencies >60 Hz tend to elicit a continuous sensation. According to pre-clinical studies (3, 4), 300 Hz stimulation even with low PW inhibits pathological slow wave brain oscillations (Figure 3C) and provides good for clinical implementation (6, 58). This may be noticed when stimulation is delivered to higher thoracic levels, where the cord has a small diameter and is relatively close to the dural membrane (Figure 4B). A drawback of continuous stimulation at higher frequencies is high-energy consumption, which makes the generator recharge intervals quite short.

More recently, the technological progress in electrode construction provided a larger number of contacts (up to 32) with intelligent programming software. To date, percutaneous paddle leads have not been tested for PD symptoms and gait problems. Novel implantable pulse generators provide SCS systems with multiple programming platforms, such as frequencies of up to 10 KHz and burst waveforms intermingled with pauses that allow paresthesia free stimulation. This type of stimulation will be very useful in blinded studies.

### Relevant Data From Clinical Studies

Spinal cord stimulation (SCS) has been used to treat refractory pain for over 50 years. Since the 1970s, several reports have been published showing the motor benefits of this technique (82–84). The pioneer paper by Cook using SCS in patients without pain was published in 1973 (85). He described five patients with multiple sclerosis treated with high frequency SCS at the upper thoracic cord who had a major improvement on disability caused by pyramidal, cerebellar, and brain stem symptoms (85). Subsequent reports have then been published using SCS to treat a wide range of motor disorders, including spasticity (86), spasmodic torticollis (84, 87), and orthostatic tremor (88, 89). In 1997, Waltz published a review of 1,336 cases with multiple sclerosis, cerebral palsy, spinal cord injury, dystonia, spasmodic torticollis, spinocerebellar ataxia, and post-traumatic brain injury who had marked or moderate amelioration after SCS (63). Particular improvements in balance, stability, gait and posture were noted.

After the initial experience described above, SCS for movement disorders has reemerged in the past decade following the spark generated by preclinical reports (3, 4).

#### Anecdotal Reports

At first, investigators described the effects of SCS in patients with PD that also had refractory pain and postural inclination (5). Although we recognize the importance of the following reports, the absolute results should be analyzed with caution due to the fact that the overall improvement in gait may also be related to an improvement of other conditions (e.g., pain) and also that none of them included placebo arm or trial. Those studies showed no significant improvement in FOG but gait and balance were not considered as primary outcome measures (90). Fénelon et al. presented the case of a 74 years old patient who developed PD 8 years after T9–T10 SCS for failed back syndrome (72). The authors objectively demonstrated improvements in tremor, bradykinesia, and rigidity with stimulation at 130 Hz. No benefit was found on gait, as measured by time to walk 7 m, turn, and walk back (72). In contrast, Landi et al. described an improvement in gait and postural instability after T9–T10 stimulation in a chronic pain patient with PD previously treated with STN-DBS (73). Hassan et al. described a 43-year-old PD patient with progressive improvement in the timed 10-m walk test and UPDRS part III 2 years after SCS implanted in the C2 region for neck and upper extremity pain (74). Akiyama et al. showed improvement on timed up and go test and camptocormia 29 days after SCS implantation at the level of T8 in a 65-year-old PD patient previously treated with bilateral STN-DBS (75). Soltani and Lalkhen presented serendipity results of improvement in leg tremor and other unrated parkinsonian symptoms (76). A common feature of these open label reports is that stimulation parameters and the spinal level of electrode implantation were apparently defined based on routine SCS protocols for pain. As such, percutaneous leads were largely implanted in lower thoracic levels and stimulation delivered at wide pulse width and frequencies that ranged from 7 to 130 Hz. An improvement in parkinsonian features was unexpectedly observed in those patients, but fortunately reported. No specific tests were done to establish optimal parameters to treat motor symptoms. More recently, Kobayashi et al. (77) described a PD patient with intractable pain treated with thoracic SCS (T6–T8) who had substantial improvements in motor scores (70%), posture and gait measures (25% sagittal vertical axis; 25% time, and 28% number of steps in the 20 m walking test). An interesting aspect of that study is that, in addition to tonic stimulation, the patient received burst SCS with no associated paresthesias. While both therapies were found to be effective, less amplitude was required for a good post-operative outcome when burst stimulation was delivered (40 Hz burst with five spikes of 500 Hz). Although those reports suggested a benefit on walking, the improvement in pain was still a major confounder, as stated by Thiriez et al. (91).

#### Clinical Studies Primarily Focusing on SCS for Axial Symptoms and Gait

The series of studies described above encouraged further trials using SCS in PD and the development of protocols to specifically assess motor outcomes (Table 1). Thevathasan et al. (92) investigated the effect of high cervical SCS in two PD patients who were blindly evaluated while receiving suprathreshold and subthreshold stimulation at frequencies as high as 130 Hz. Overall, no improvements in UPDRS motor score and gait assessment were noticed.

**Table 1:** Studies approaching SCS as a treatment of motor symptoms and gait disorders in Parkinson's disease.

**References**	**No. of patients**	**Mean disease duration (years)**	**DBS prior to SCS**	**Electrode type**	**SCS level**	**Freq**	**PW**	**Kind of stimulation**	**Dopa condition at evaluation**	Evaluations/ Follow up (months)	**Study design**	**UPDRS motor score: improvement(%)**	**Gait Analyses**	**Other outcomes: Improvement (%)**
Thevathasan et al. (92)	2	NA	No	Quadripolar and octopolar; cylindrical	High cervical	130 and 300 Hz	240 and 200 μs	Tonic Supra threshold and sub threshold for each patient	Night withdrawal	10 day PO/None	Acute double blind crossover between two conditions (supra and sub threshold) with a washout of 20 min.	0% 0%	Timed 10 m walk: no improvement	Timed hand arm movements: 0% Timed lower limb tapping:0%
Agari et al. (78)	15	17.2	Seven cases	Quadripolar and octopolar; cylindrical	T7–T12	5–20 Hz	210–330 μs	NA	On med	Baseline, 3 and 12 months/12 months	Case series (prospective)	19.5% at 3 months 9% at 12 months	Timed 10 m walk: improvement of 9.2% at 3 and 2.1% at 12 months. TUG: improvement of 25.7% at 3 and 13.3% at 12 months.	Postural improvement at 3 months 25%; at 12 months 9%
Pinto de Souza et al. (6)	4	21.2	Four cases (mean 7.8 years before SCS)	Three columns (5-6-5); paddle	T2–T4	300 Hz	90 μs	Tonic 105% of the threshold for paraesthesia	12 h withdrawal	Baseline, 1, 3, and 6 months/6 months	Case series (prospective)/ Blinded randomized evaluation with 60 × 300 Hz at the 4 month with a washout of 2 h between conditions.	36.8% at 1 month 48.7% at 3 months 38.3% at 6 months	20 m walk: improvement of 58% on time and 65.7% on steps numbers at 6 months. TUG: improvement of 63.2% at 6 months. TUG with double task: improvement of 54% at 6 months. Stride length: increase of 170% at 6 months.	PDQ 39: improvement of 44.7% at 6 months. FOG: improvement of 56.4% at 6 months.
Samotus et al. (7)	5		No	Double octopolar; cylindrical	T8–T10	30–60 Hz	200–500 μs	Tonic Supra threshold for paraesthesia	On med	Baseline, 4, 6 months/6 months	Case series (prospective)/11 frequencies and pulse width different combinations for each patient.	33.4% at 6 months	Stride length: increase of 38.9% at 6 months. Steps velocity: increase 29.4% at 6 months. Swing improved 21% at 6 months.	FOG: improvement in 26.8%. at 6 months. ABC (daily activities): improvement of 65% at 6 months.
Kobayashi et al. (77)	1	3	No	Double octopolar; cylindrical	Th6–Th8	Burst DR high frequency		Burst No paraesthesia	NA	14 days after Burst SCS/None	Case report	70% after 14 days	20 m walk: improvement of 25% on time and 28% on steps numbers.	Sagittal vertical axis improvement of 25%.
de Lima-Pardini et al. (58)^*^									12 h withdrawal	Three conditions (blinded randomized): SCS 300 Hz frequency; SCS 60 Hz frequency; 3) SCS off				300 Hz SCS improved APA (time and amplitude) and reduced time of Fog.
Hubsch et al. (8)	5	14.8	1 patient (no details)	Octopolar; cylindrical	Th 10-Th 11	100 Hz	300 μs	Tonic Supra threshold for paraesthesia	On /Off Med	60 days	Case series (prospective)/ Short Follow up	On SCS 23% On Med +On SCS 36.8% After 60 days	Stand-walk-sit test On SCS 23.6% On Med +On SCS 29.8%	FoG-Q–no improvement PDQ39–small improvement

* *This study was an extension of study 3*.

Agari et al. (78) implanted thoracic SCS electrodes (T7–T12) in a series of 15 patients with moderate to advanced PD suffering from refractory back and leg pain. At 3 months, UPDRS motor scores improved by 19.5%, measures of daily life activity by 21%, timed 10 m-walk by 9.5%, timed up and go test by 25.7%, and postural scores by 25%. However, the magnitude of these beneficial effects declined by 12 months with significant results still being detected only for TUG (13.3% compared to baseline). No control group was proposed in this study and, as stated above, the presence of pain and especially its improvement may be pointed as bias.

Pinto de Souza at al. (6) have implanted high thoracic SCS electrodes (T2-4) in four DBS-treated PD patients with prominent gait dysfunction. Implant site, electrode geometry (paddle leads) and stimulation settings (300 Hz/90 μs) were similar to those used in animals models. At 6 months, UPDRS motor scores improved by 38.3% while various gait parameters were improved by 54–65%. There were also improvements in quality of life (PDQ 39 by 44.7%) and FoG (FoG-Q questionnaire by 56.4%), suggesting steady clinical progress. To test the possibility of a placebo effect and bias associated with SCS-induced paraesthesias, a blinded randomized crossover evaluation was conducted comparing off stimulation, 60 and 300 Hz on the fourth month of treatment. While 300 Hz significantly improved gait measures, in average SCS at 60 Hz was not as effective. This is of particular importance in times of skepticism as to whether SCS is effective, especially when FoG is considered. The same group of patients was studied in a gait laboratory (1) to address the effects of SCS on FoG and distinct domains of postural control, including APA. The gait behavior was assessed through kinematics and kinetics, which allowed for objective outcomes, mainly for the assessment of the occurrence and duration of FoG, and amplitude and time of APA. For the first time FoG was objectively evaluated during SCS using a recent frequency domain approach to determine FoG events (93).

As for clinical observations, although both SCS at 300 and 60 Hz improved APA and the duration of FoG episodes in relation to the OFF-SCS condition, SCS at 300 Hz showed significantly higher benefits than 60 Hz. The duration of FoG after 60 Hz SCS improved by 73% compared to 91% after 300 Hz. The time of APA improved by 4.35% after 60 Hz SCS and 17% following 300 Hz stimulation. In contrast, reactive postural control was not affected by SCS.

Samotus et al. (7) studied five male PD patients treated with SCS delivered through percutaneous electrodes implanted in lower thoracic levels. Although patients were followed overtime, no double-blind trial was described in this report. Optimal stimulation parameters were selected over different frequencies (range 30–60 Hz) and broad pulse widths (200–500 μs). The authors observed acute decreases in FoG episodes during at least two evaluation sessions in the laboratory to objectively assess gait parameters (velocity, stride length, swing), always under the effect of levodopa. Improvements in UPDRS motor scores (33%), Activities-specific Balance Confidence (ABC) daily activities (65%), swing (21%), stride length (38.9%), velocity (29.4%), and FoG (26.8%) were observed during acute evaluation sessions at 6 months. Of note, the best reported results were observed when high pulse widths were used. This fact is quite interesting because it corroborates the concept that larger pulse widths tend to be less selective, as less excitable neuronal elements also tend to depolarize. In the same direction, electrical current has also been considered to reach further deep into the spinal cord. In the lumbar spinal cord enlargement, stimulation would theoretically require larger pulse widths to reach a wider range of ascending systems, as the cord diameter is considerably wider. On the frequency side, data from this series does not specify if SCS at 300 Hz was tested, as described in the pre-clinical study by Fuentes et al. (4) and clinical data from the study by Pinto de Souza et al. (6). One possible explanation might be related to the different stimulation site in the lower cord. This apparent diversion needs to be further studied.

More recently Hubsch et al. (8) have studied five PD patients with prominent axial symptoms who received monopolar stimulation (100 Hz/300 μs) from a single midline percutaneous epidural lead at the level of T10–T11. Patients were assessed OFF and ON levodopa at short term (60 days). Though a blinded evaluation of videos was conducted for the stand-walk-sit test, patients could still feel the paresthesias when SCS was ON. In average, patients performed better during gait assessments with ON-SCS + ON-Ldopa. Improvements with SCS (23.6%) or levodopa (19.3%) were similar with a synergistic effect recorded when both therapies were administered in conjunction (29.8%). Similar effects were observed in the MDS-UPDRSIII; While the improvement with ON-SCS (23.22%) did not differ from ON-Ldopa's, ON-SCS + ON-Ldopa led to a 36.8% improvement. No significant changes were observed in FOG-Q but PDQ39 improved slightly, especially in the mobility scores at 60 days. The positive effects observed in this series were accomplished with 130 Hz stimulation and a large pulse width (300 μs). The remaining parameters and stimulation site were similar to Samotus at al. (7).

Freezing of gait (FoG) and gait disturbance are not exclusively observed in PD but also in atypical parkinsonism. Rohani et al. described two patients with primary progressive FoG treated with SCS at T10–T11. Gait analyses revealed an improvement in FOG and gait at 5 and 24 months, respectively (93). Unrelated to FoG or PD, a recent series of studies have shown promising results with the use of SCS to treat motor deficits in patients with spinal cord injury (94, 95).

The above-reviewed reports suggest that cardinal symptoms of PD can improve following SCS. Of particular interest, however, would be locomotion improvements in patients with gait problems, especially FoG. Most PD symptoms respond well to medication alone and additional deep brain stimulation (DBS) (96). Even FoG may improve chronically with DBS when this symptom responds acutely during the levodopa challenge test (97). So, FoG subtype unresponsive to medication or DBS may in the future be one of the indications to SCS in PD. According to the report of de Souza et al. (6), patients with advanced PD chronically treated with DBS who develop unresponsive FoG despite effective treatment to other symptoms, also benefit from SCS. Yet, PD patients who somehow cannot receive DBS may be another indication for SCS, once cardinal symptoms and gait problems respond (7, 8, 78). On the other hand, SCS does not seem to potentiate levodopa, as observed with subthalamic nucleus DBS, or to block dyskinesias, as commonly described following internal pallidum stimulation (6, 7). A word of caution should be added to the comments above because most of the data disclose in the literature does not include control arms (6) and are considered low class evidence. Well-designed trials including double blind and placebo control arms with a large sample size and specific stimulation protocols are still needed for SCS to be considered as a potential treatment.

## Final Remarks

The therapeutic use of SCS in patients with movement disorders is not novel. However, the field was recently rekindled by preclinical experiments providing a stronger rationale, optimized stimulation settings, and better appraisal of potential mechanisms (3, 4). Clinical trials following some aspects described in those studies have recently been conducted with promising results. With accumulation of experience and based in a more comprehensive amount of data, the importance of a few aspects became clear. *Choice of electrode*. The electrical field created by single cylindrical and paddle electrodes is fairly different. Paddle electrodes require a surgical approach while cylindrical electrodes can be implanted percutaneously. The former, however, covers a wider portion of the spinal cord and allows several configurations that may modulate different tracts and neural elements. *Choice of generator*. Currently, generators that provide stable energy delivery by automatic positional control and new generators that allow burst and kHz stimulation could facilitate the design of blinded studies, since no paresthesias are felt. *Spinal level*. While benefits were shown following cervical and low thoracic stimulation, a more comprehensive analysis with data from animal studies and translational clinical implementation suggests that the upper thoracic cord may be the hot spot for SCS. Stimulation of the cervical and lumbar spinal cord enlargements has also been described. *Stimulation parameters*. The most effective electrical wave type may be different for each spinal cord level, but apparently they all point to the need of recruiting less excitable elements, including those deep-seated in the spinal cord. In addition to electrode configuration, defining appropriate pulse width, and the frequency most suited to treat different PD symptoms would be important to optimize the therapy and standardize studies. *Clinical characteristics of the treated population*. It is important to define the clinical phenotype and symptoms that better respond to SCS, as well as stimulation interactions with medication regimens, including L-DOPA.

Based on the information gathered and summarized above, we expect the future development of well-designed trials including specific disease phenotypes. If FoG is the intended condition to be treated, experienced clinical staff should be involved, since this is an episodic phenomenon highly influenced by internal and external factors. In one hand, gait lab evaluations are important to calculate the metrics of gait change. However, lab settings can cause biases in the determination of outcomes. Only part of the outcome measures should take place in gait labs. Data should also be generated in conditions as close as possible from every day life conditions. In addition, measurements capable of identifying changes in locomotion, the occurrence and severity of FoG episodes and other disabilities, such as falls, should be included. Other methods to obtain information in longer periods as functional scales, diaries or actigraphic monitoring should also be considered, since they provide additional information to the ones obtained in gait labs. Visits should be short to avoid testing too many experimental conditions at the same time because patients can get tired in long sessions and recorded information may not be accurate. The design of trials should include few test conditions and sufficient time for the wash out between interventions, including the surgical procedure itself; all patients should endure this period after implantation. Surgical procedures can induce a strong placebo effect, which in FoG should be seriously considered. If possible, a method for blinding patients and observers should also be included in order to reach the highest level of evidence. Adapting the technology and procedures for each particular neurological condition and severity will hopefully provide stronger data and establish indications for the used of SCS in conditions associated with FoG.

## Author Contributions

AdL-P, DC, BMo, and CP wrote the first draft of the article. EF, CH and AdL-P wrote the final draft of the article. MdS, CH, and EF provided the contextual frame of the review. All authors critically revised the manuscript. EF, AdL-P, and DC designed the figures.

### Conflict of Interest Statement

The authors declare that the research was conducted in the absence of any commercial or financial relationships that could be construed as a potential conflict of interest.
